# Long-Term Follow-Up Of Anti-Mullerian Hormone Levels After Laparoscopic Endometrioma Cystectomy

**DOI:** 10.7150/ijms.69830

**Published:** 2022-03-21

**Authors:** Nguyen Duy Anh, Nguyen Thi Thu Ha, Nguyen Manh Tri, Do Khac Huynh, Do Tuan Dat, Phan Thi Huyen Thuong, Nguyen Khac Toan, Tran Anh Duc, Nguyen Duc Hinh, Hoang Van Tong

**Affiliations:** 1 Department of Gynecological of Hanoi Obstetrics and Gynecology Hospital, Hanoi, Vietnam; 2 Department of Obstetrics and Gynecological of Hanoi Medical University, Hanoi, Vietnam; 3 Department of assisted reproduction of Hanoi Obstetrics and Gynecology Hospital, Hanoi, Vietnam; 4 Institute of Biomedicine and Pharmacy, Vietnam Military Medical University, Hanoi, Vietnam

**Keywords:** endometrioma, anti-mullerian hormone, cystectomy, cyst size

## Abstract

**Objective:** The study aims to evaluate long-term ovarian reserve change by serum anti-Mullerian hormone (AMH) level and determine the factors that affect the changes after laparoscopic endometrioma cystectomy.

**Methods:** In a prospective longitudinal study, 104 patients with unilateral (n=77) and bilateral (n=27) endometrioma underwent laparoscopic endometrioma cystectomy. AMH levels were measured preoperatively and at 1, 3, 6, and 12 months postoperatively. Multivariate linear regression analysis was performed to determine factors related to AMH level changes.

**Results:** Median preoperative AMH levels significantly declined from 3.77 ng/mL to 1.60 ng/mL (*P*<0.001), 1.66 ng/mL (*P*<0.001), 1.67 ng/mL (*P*<0.001), and 1.72 ng/mL (*P*<0.001) at 1, 3, 6, and 12 months postoperatively, respectively. The rate of decrease in AMH was unchanged six months after surgery, 52.2%, 53.7%, 54.8% at 1, 3, 6 months, respectively, and declined to 43.2% at 12 months. Although most factors were associated with AMH level changes in monovariant linear regression, multivariant linear regression analysis showed only three factors that reached the statistical significance, including bilateral endometriomas, mean size of the endometrioma, and preoperative AMH levels.

**Conclusions:** Serum AMH levels decline significantly after laparoscopic cystectomy of endometriomas but recovered at 12 months compared with the first 6 months with unilateral endometrioma. Bilateral endometriomas, size of the cyst, and preoperative AMH levels might independently affect AMH levels at 12 months after surgery.

## Introduction

Endometriosis is a common gynecological disease, accounting for an incidence of 10% in women of reproductive age, and up to 50% of infertile women, with the main clinical manifestations being pain and infertility. Endometriomas are one of the most common forms of endometriosis, with a prevalence as high as 17%-44% in patients with endometriosis and approximately 35% in those with benign ovarian tumors 1. Although the most effective and widely used treatment method is laparoscopic endometrioma cystectomy because of a treatment goal of pain relief, the increased likelihood of spontaneous pregnancy and reduced progression and recurrence of the disease 2-5, this surgical intervention causes loss of follicles thereby affecting ovarian reserve and fertility 6-8. Therefore, the surgical solution is still a controversial treatment for endometriomas.

There are several tests used to evaluate ovarian reserve. However, until now, anti-Mullerian hormone (AMH) and antral follicle count (AFC) are proposed as the two most valuable, of which AMH is considered more advantageous than AFC. AMH has the earliest predictive value, is independent of the menstrual cycle, and is not affected by oral contraceptives, gonadotrophin-releasing hormone agonists, endometriosis, or a history of ovarian surgery 9, 10. Therefore, AMH is often the preferred choice to evaluate ovarian reserve after endometrioma cystectomy.

Several studies of the change in ovarian reserve by AMH after laparoscopic endometrioma cystectomy have been published 11-20. The systematic review and meta-analysis by Raffi F et al. indicated a significant reduction in AMH levels after surgery 11. However, the studies had small sample sizes, short follow-up times, and unclear related factors, thus more definitive studies are needed to determine the change in longitudinal AMH levels 10. Recent longer follow-up studies showed different results, some of which observed AMH levels are unchanged six months after surgery 12-15, other studies indicated that AMH could recover one year after surgery 16-20.

The current study aimed to evaluate long-term ovarian reserve change by serum AMH levels to understand whether the decrease of AMH levels is persistent or recovered after surgery and to determine pre- and intra-operatory factors that affect the changes after laparoscopic endometrioma cystectomy.

## Material and methods

### Study subjects

This prospective study was carried out from 2015-2017 at Hanoi Obstetrics and Gynecology hospital, Hanoi, Vietnam and was approved by the Ethics Committee of the Hospital. Inclusion criteria were as follows: reproductive age 18 to 40 years with regular menstrual cycles, and had unilateral or bilateral endometrial cysts with no suspected malignancy on ultrasound. Exclusion criteria were endometrioma with another ovarian cyst, history of previous ovarian surgery, unconfirmed endometriosis or suspected malignancy on histology, hormone therapy used within three months before surgery, or any endocrine disorder including polycystic ovarian syndrome, hyperprolactinemia, thyroid dysfunction, and Cushing's syndrome.

### Procedures

Eligible women were informed about the study by gynecologists and written informed consent were received from all participants before enrolling in this study. Before surgery, baseline obstetric and medical histories were recorded. Cyst size was measured through transvaginal ultrasound by calculating the average of 2 diameters: a large diameter and a small diameter perpendicular to the large diameter. The mean size of endometriomas was calculated as the size of the total cyst divided by the number of endometriomas. Pain score was assessed by the Numerical Rating Scale (NRS). CA125 levels were measured in all participants by the Cobas E602 Immunology Analyzer (Roche, Basel, Switzerland) based on the electro-chemiluminescence immunoassay.

The surgical technique was stripping technique with the main procedure consisting of scissors or unipolar coagulation to open the capsule. The endometrioma was removed from the normal ovarian tissue attempting to avoid rupture. Hemostasis of normal ovarian tissue was controlled by bipolar coagulation using water to find bleeding points and stop them. The operation duration was calculated from when the surgeon started to fully insert the laparoscopic instruments into the abdomen to when the trocars began to be withdrawn. The revised American Society for Reproductive Medicine (rASRM) classification was used to determine the score and endometriosis stage 21. All resected specimens were diagnosed by histology.

### Follow-up

Immediately after surgery, a 3-month course of Gonadotrophin-releasing hormone agonists (GnRHa) or a 6-month course of combined oral contraceptives (COCs) were administered to 10 and 37 patients, respectively. The selection of patients was based on the grade of endometriosis and the desire for pregnancy. No medications were used in 57 patients for postoperation medical treatment. Serum AMH levels were measured at 1, 3, 6, and 12 months after surgery. No pregnancy was recorded within the first 6 months. Nineteen (n=19) patients conceived spontaneously from 6 to 12 months who were without measured serum AMH levels at 12 months.

### Hormonal assays

Serum AMH levels were measured the day before surgery and at 1, 3, 6, and 12 months postoperatively by a fully automated anti-Müllerian hormone assay (Access 2 IA AMH assay; Beckman Coulter) with a detection limit of lower than 0.02 ng/mL. The rate of decrease in AMH level (dAMH) was calculated for each patient using the formula as follows: 100 x (preoperative AMH level - postoperative AMH level) /preoperative AMH level. In addition, Bologna criteria with AMH levels <1.1 ng/mL are used to assess diminished ovarian reserve (DOR) 22.

### Statistical analysis

Quantitative variables were tested for normal distribution by Skewness and Kurtosis tests. With non-normally distributed variables, mediant and interquartile ranges were used in descriptive statistics. The Mann-Whitney test was applied to compare the groups. The Wilcoxon signed-rank paired test was used to determine the differences in serum AMH levels between each sampling point and the rate of decrease in the AMH levels. The relationship between two quantitative variables was established by Spearman's test. Multivariant linear regression analysis was used to explore the factors affecting post-operative AMH levels. The Stata (version 14.0) software was for analysis. Statistical significance was set to a *P*-value of <0.05.

## Results

The characteristics at baseline of the 104 patients undergoing laparoscopic endometrioma cystectomy in this study are described in table 1. Seventy-seven patients (74.04%) had unilateral endometriomas, and 27 patients (25.96%) had bilateral endometriomas. The median size of endometriomas was 5.9 cm and forty-eight patients (45.15%) had infertility. The median serum AMH level was 3.77 ng/mL and all the patients were in stage III-IV of endometriosis according to rASRM (Table 1).

**Figure 1:**
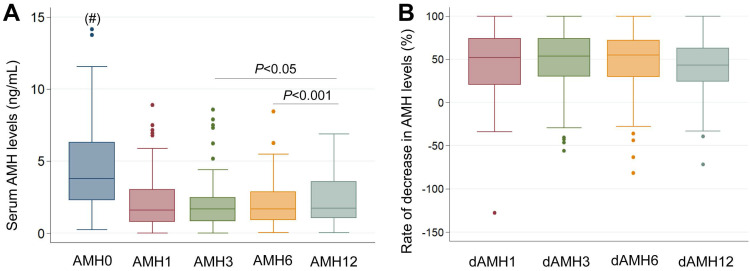
** Longitudinal follow-up of AMH levels after laparoscopic endometrioma cystectomy.** (A) Median AMH levels assessed before and after surgery. AMH0: Serum AMH levels before surgery; AMH1, AMH3, AMH6, AMH12: Serum AMH levels at 1 month, 3 months, 6 months, and 12 months after surgery, respectively, (#) *P*<0.001 when compared with other groups. (B) Rate of decrease in AMH levels (dAMH) after surgery. dAMH1, dAMH3, dAMH6, dAMH12: rate of decrease in AMH levels (dAMH) at 1 month, 3 months, 6 months, and 12 months after surgery, respectively.

The primary outcome of the study was to evaluate long-term ovarian reserve change by serum AMH levels. The median of preoperative AMH levels significantly declined from 3.77 ng/ml to 1.60 ng/mL (*P*<0.001), 1.66 ng/mL (*P*<0.001), 1.67 ng/mL (*P*<0.001), and 1.72 ng/mL (*P*<0.001) at 1, 3, 6, and 12 months postoperatively, respectively. However, the AMH levels at 12 months were significantly higher compared to those at 3 months and 6 months (*P*<0.05). The rate of decrease in AMH was 52.2%, 53.7%, 54.8% at 1, 3, 6 months after surgery compared to that of baseline levels, respectively. However, the rate of decrease in AMH was lower at 12 months (43.2%) than in the first six months after surgery (*P*<0.05) (Figure 1 and Table 2). In addition, the rate of decrease in AMH levels was different between unilateral and bilateral endometriomas. While the rate of decrease was 48.0%, 48.2%, 43.4%, and 34.9% in unilateral endometriomas, it was 78.0%, 85.0%, 83.6%, and 83.2% in bilateral endometriomas at 1, 3, 6, and 12 months postoperative, respectively. In bilateral endometriomas, the rate of decrease in AMH level was unchanged for 12 months postoperative, but in unilateral endometriomas, it was unchanged within the first 6 months and lower at 12 months compared to the first six months postoperative (Table 2).

**Figure 2:**
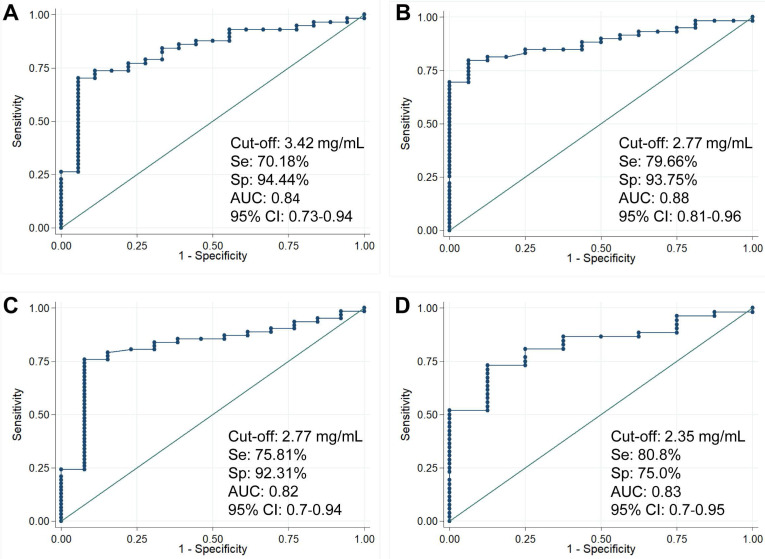
** Pre-operative AMH level cut-off to predict diminished ovarian reserve after surgery.** The cut-off values of AMH levels before surgery to predict diminished ovarian reserve (DOR) 1 month (A), 3 months (B), 6 months (C) and 12 months (D) after surgery in patients with unilateral endometriomas. Cut-off values, sensitivity, specificity, area under the curve (AUC) and 95%CI are presented on the figures.

We have analyzed whether the cut-off values of preoperative AMH levels could predict diminished ovarian reserve (DOR) after surgery. In the group with unilateral endometriomas, the results showed that AMH levels before surgery could predict diminished ovarian reserve (DOR) 1 month, 3 months, 6 months and 12 months after surgery with high prognostic performance (AUC: 0.84, 0.88, 0.82 and 0.83, respectively). Particularly, the cut-off value of pre-operative AMH levels of 2.35 mg/mL could predict diminished ovarian reserve (DOR) 12 months after surgery with 80.8% sensitivity and 75.0% specificity (Figure 2). However, no significant association of preoperative AMH levels with diminished ovarian reserve (DOR) was observed in the bilateral endometriomas group.

The secondary outcome of the study was to determine the factors that may affect the changes after laparoscopic endometrioma cystectomy. Several factors were associated with AMH levels postoperatively. Those factors included age, pain score, laterality, size of the cyst, pre-operative AMH level, duration of operation, score, and stage of endometriosis (Table 3). Multivariant linear regression analysis showed that only three factors were associated with AMH levels, those being bilateral endometriomas, endometrioma size, and pre-operative AMH level (Table 4). In addition, this study recorded 19 patients with spontaneous pregnancy within 6 to 12 months postoperative, of which 14 patients were infertility (29.2% infertility patients) and five patients desired to have more baby.

## Discussion

Laparoscopic endometrioma cystectomy is a recommended and widely used method because it meets the treatment goals of endometriosis, which is to reduce pain, increase the chance of spontaneous pregnancy, and reduce disease progression and recurrence 2-5. However, haemostasis of the cyst bed during surgery can cause follicle loss, which can affect ovarian reserve. Thus, coagulation should be kept to a minimum, especially for patients with reproductive goals 2-5. A significant decrease in serum AMH levels after laparoscopic endometrioma cystectomy has been documented 12, 14, 16-19. A systematic review and meta-analysis study showed that the median AMH level before surgery was 3.1 ng/mL and significantly decreased to 1.51 ng/mL within one to nine months postoperative 11. Some studies with 30 patients (six-month follow-up) 23 and with 25 patients (three-month follow-up) 24 showed no difference in serum AMH levels between pre and post-operation. Our study with 104 patients showed that preoperative AMH levels significantly declined from 1, 3, 6, and 12 months postoperatively, respectively. The decrease in serum AMH levels was higher in the bilateral endometriomas group compared with the unilateral endometrioma group. These results were similar to those found in a systematic review and meta-analysis 25. Serum AMH levels were persistent or recovered after surgery, and long-term ovarian reserve changes by serum AMH levels were evaluated. The time point and duration of follow-up was different amongst studies. Most of the studies involved follow-up to six months after surgery 12, 13, 23 while Alborzi S et al. followed up at 9 months after surgery 14. To the best of our knowledge, our present study is one of the longest follow-up studies with a duration of 12 months after surgery 16-19, 26.

Most studies with 6-month follow-up showed that AMH levels decreased significantly and were persistent within 6 months post-operation 12-15. The study by Vignali M et al. showed that AMH levels decreased one month after surgery but increased gradually after 3 and 6 months of surgery 17. The longitudinal studies of 12 months determined serum AMH levels could recover after surgery 16-19. However, it was different when the recovery was compared between AMH levels at 12 months post-operative and pre-operative or previous timepoint postoperatively, and the recovery of AMH levels at 12 months post-operatively was equivalence with the pre-operative levels or improved compared with a previous timepoint after surgery. Although AMH levels have been shown to be significantly decreased one month after surgery in all patients, nearly half of those had higher post-operative AMH levels at one year compared to one month 16. Studies observed that AMH levels completely recovered 12 months after surgery compared to preoperatively and that the decrease in AMH levels after surgery was only temporary and that full recovery after surgery was possible 17, 18. Similarly, another study showed that although ovarian reserve significantly decreased after surgery, serum AMH levels could recover at 12 months compared to six months in the unilateral endometrioma group but were consistent in the bilateral group 19. However, ovarian reserve did not significantly decrease in the long term with 30.4±18.0 months follow-up after endometrioma surgery 20. In line with most previous studies, we indicated that in the unilateral endometrioma group, the AMH levels post-operation were significantly decreased compared with pre-operative, but unchanged within the first six months and recovered at 12 months compared with previous timepoints after surgery (1, 3 and 6 months). A significant decrease in AMH levels after surgery was also observed in the bilateral endometrioma group; in contrast, there was no recovery of AMH levels at 12 months postoperatively.

Our result indicated that preoperative AMH levels could be a biomarker to predict diminished ovarian reserve (DOR) after surgery. Based on Bologna criteria 22, women with a diagnosis of poor ovarian response after laparoscopic cystectomy endometriomas have significantly lower pregnancy and live birth rates compared to those with primary poor response 27. Therefore, finding out the biomarker such as preoperative AMH levels that could predict diminished ovarian reserve (DOR) after surgery is important to choose appropriate treatments to preserve fertility in infertile patients with ovarian endometriomas. The hypotheses presented to explain the recovery of AMH after surgery have been documented 16, 28. After surgery, the blood damaged vessels of the ovary will regenerate to nourish the ovary restoring AMH levels. The function of granulosa cells in the remaining follicles is stimulated so that although the number of follicles is reduced, the amount of AMH produced may increase. The remaining follicles can be recovered from "silent follicles" to "active follicles" thus the ovarian reserve is partially restored: laparoscopic surgery with partial burning of ovarian tissue can stimulate follicular differentiation from the surface epithelium or myeloid cells, however, this hypothesis is controversial. In addition, the delay in the recovery of AMH levels might be explained by the approximate 180-day duration of folliculogenesis.

There have been many studies looking for factors affecting ovarian reserve change after laparoscopic endometrioma cystectomy. However, most studies used univariate analyses, many factors were believed to influence the AMH levels. The impact factors include laterality, pre-operative AMH levels, age and size of the cyst 12, 14, 23, 29-31. Our study using the univariate analyses indicate factors that affected AMH levels postoperatively including age, pain score, laterality, cyst size, pre-operative AMH levels, duration of surgery, rASRM score and endometriosis stage. Therefore, multivariate analyses are necessary to determine which factors affect the change of ovarian reserve after laparoscopic endometrioma cystectomy. By including all affected factors in univariate analysis into multivariable analysis, our results determined three predictive factors of AMH levels at 12 months post-operative, including pre-operative AMH levels, laterality of endometriomas, and the size of the cyst, in which laterality of endometriomas had the greatest weight in the decrease of AMH levels with the largest beta coefficient. To date, not many multivariate analyses were performed to examine factors affecting changes in ovarian reserve after laparoscopic endometrioma cystectomy 18, 19, 32. By performing multivariate analysis, earlier studies indicated that the affecting factors could be laterality and duration of surgery 32; patients age, baseline AMH levels, and bilateral endometriomas 19; bilateral cyst, size of the cyst, and stage of endometriosis 18.

Increasing the chance of spontaneous pregnancy is one of the advantages of laparoscopic endometrioma cystectomy 2-5. In this study, among 48 patients who did not use any additional fertility treatments within 12 months after surgery, 14 (29.2%) infertile patients reported having spontaneous pregnancy. Also, our data are important for preoperative counselling, regarding individual reproductive plans for women with endometriomas. Although the current study has some advantages such as a larger number of patients, longer follow-up period, and performing multivariate analysis to find predictors of post-operative AMH levels, limitations are remaining. The most important limitation is that the control group was not included. More studies on surgical techniques and hemostasis methods as well as longer follow-up time should be conducted in the laparoscopic endometrioma cystectomy method.

In conclusion, our study showed that serum AMH levels decline significantly after laparoscopic cystectomy of endometriomas and recover at 12 months compared with the first 6 months with the unilateral endometrium. The factors that may affect AMH levels at 12 months after surgery are bilateral endometriomas, mean size of the cyst and preoperative AMH levels.

## Figures and Tables

**Table 1:** Baseline characteristics of the study subjects

Characteristics	Values
**Age (years)**	28.5 (25-34)
Median (Interquartile)	
**BMI**	19.83 ±2.12
Mean ±SD	
**PreNRS**	5 (3-7)
Median (Interquartile)	
**Infertility**	
Yes (n, %)	48 (45.15)
No (n, %)	56 (53.85)
**Laterality**	
Unilateral (n, %)	77 (74.04)
Bilateral (n, %)	27 (25.96)
**Cyst size (cm)**	5.9 (4.9-8.1)
Median (Interquartile)	
**CA125 (UI/mL)**	69.15 (0.95-106.9)
Median (Interquartile)	
**PreAMH (ng/mL)**	3.77 (2.28-6.31)
Median (Interquartile)	
**rASRM Score**	37 (25-60)
Median (Interquartile)	
**Endometriosis Stage (n (%)**	
Stage III (n, %)	56 (53.85)
Stage IV (n, %)	48 (46.15)
**Duration of operation (minutes)**	45 (35-60)
Median (Interquartile)	

BMI: Body mass index; NRS: Numerical Rating Scale; CA125: cancer antigen 125; AMH: Anti-Mullerian Hormone; rASRM: revised American Society for Reproductive Medicine.

**Table 2:** Serum AMH levels in patients with unilateral and bilateral endometriomas

Endometriomas	Baseline	1 month	3 months	6 months	12 months (n=85)
**All patients**					
**AMH (ng/mL)**					
Median (Interquartile)	3.77 (2.28-6.31)	1.60 (0.77-3.01)	1.66 (0.82-2.47)	1.67 (0.91-2.86)	1.72 (1.02-3.58)
**dAMH (%)**					
Median (Interquartile)		52.2 (20.4-74.1)	53.7 (30.1-74.1)	54.8 (29.7-71.8)	43.2 (24.1-62.9)
***P* value**					
vs. baseline		**< 0.001**	**< 0.001**	**< 0.001**	**< 0.001**
vs. 1 month			> 0.05	> 0.05	> 0.05
vs. 3 months				> 0.05	**< 0.05**
vs. 6 months					**< 0.001**
**Unilateral**					
**AMH (ng/mL)**					
Median (Interquartile)	3.53 (2.14-6.11)	1.95 (1.10-3.58)	1.91 (1.22-2.64)	1.94 (1.27-2.94)	2.39 (1.44-3.87)
**dAMH (%)**					
Median (Interquartile)		48.0 (11.7-60.7)	48.2 (28.1-64.1)	43.4 (21.2-62.8)	34.9 (19.8-46.0)
***P* value**					
vs. baseline		**< 0.001**	**< 0.001**	**< 0.001**	**< 0.001**
vs. 1 month			> 0.05	> 0.05	> 0.05
vs. 3 months				> 0.05	**< 0.001**
vs. 6 months					**< 0.001**
**Bilateral**					
**AMH (ng/mL)**					
Median (Interquartile)	4.91 (2.77-6.81)	0.40 (0.16-1.40)	0.77 (0.29-1.68)	0.89 (0.24-1.25)	0.92 (0.32-1.23)
**dAMH (%)**					
Median (Interquartile)		78.0 (72.3-96.0)	85.0 (57.1-92.9)	83.6 (69.3-91.7)	83.2 (70.1-90.2)
***P* value**					
vs. baseline		**< 0.001**	**< 0.001**	**< 0.001**	**< 0.001**
vs. 1 month			> 0.05	> 0.05	> 0.05
vs. 3 months				> 0.05	> 0.05
vs. 6 months					> 0.05

AMH: Anti-Mullerian Hormone; dAMH: Rate of decrease in AMH level. *P* value is for the comparation of AMH levels. Bold in *P* values represent *P*<0.05.

**Table 3:** Factors related to AMH levels pre- and post-operation

Factors	AMH levels at Pre-operation		AMH levels at 1 month		AMH levels at 3 months		AMH levels at 6 months		AMH levels at 12 months	
	*P*	r	*P*	r	*P*	r	*P*	r	*P*	r
**Age**	**<0.01**	-0.33	>0.05		>0.05		<0.05	-0.23	<0.01	-0.36
**BMI**	>0.05		>0.05		>0.05		>0.05		>0.05	
**PreNRS**	>0.05		**<0.01**	-0.31	**<0.01**	-0.30	**<0.01**	0.25	**<0.01**	-0.34
**Infertility**	>0.05		>0.05		>0.05		>0.05		>0.05	
**Laterality**	>0.05		**<0.001**		**<0.001**		**<0.001**		**<0.001**	
**Mean cyst size**	>0.05		**<0.001**	-0.35	**<0.001**	-0.32	**<0.001**	-0.33	**<0.001**	-0.44
**CA125**	>0.05		>0.05		>0.05		>0.05		>0.05	
**PreAMH**			**<0.05**	0.49	**<0.01**	0.51	**<0.01**	0.48	**<0.001**	0.52
**Duration of operation**			**<0.001**	-0.35	**<0.001**	-0.39	**<0.001**	-0.35	**<0.001**	-0.46
**rASRM Score**	>0.05		**<0.001**	-0.46	**<0.001**	-0.40	**<0.001**	-0.47	**<0.001**	0.52
**Endometriosis Stage**	>0.05		**<0.001**		**<0.001**		**<0.001**		**<0.001**	
**GnRHa**			>0.05		>0.05		>0.05		>0.05	
**COCs**			>0.05		>0.05		>0.05		>0.05	

BMI: Body mass index; CA125: cancer antigen 125; rASRM: revised American Society for Reproductive Medicine; AMH: Anti-Mullerian Hormone; NRS: Numerical Rating Scale; COCs: combined oral contraceptives; GnRHa: Gonadotrophin-releasing hormone agonists; Bold in *P* values represent *P*<0.05.

**Table 4:** Multivariate linear regression analyses with AMH levels measured at 12 months as the dependent variable

Factors	beta	standard error	*P* value	95% CI
**Age**	-0.014	0.008	0.076	(-0.029, 0.001)
**PreAMH**	0.109	0.014	**0.0001**	(0.082, 0.136)
**Laterality**	-0.763	0.125	**0.0001**	(-1.012, -0.514)
**Mean cyst size**	-0.078	0.029	**0.01**	(-0.136, -0.019)
**PreNRS**	-0.015	0.018	0.414	(-0.051, 0.021)
**Duration of operation**	0.000	0.003	0.993	(-0.006, 0.006)
**rASRM Score**	-0.002	0.003	0.394	(-0.008, 0.003)
**Endometriosis Stage**	0.045	0.124	0.717	(-0.203, 0.293)

rASRM: revised American Society for Reproductive Medicine; AMH: Anti-Mullerian Hormone; NRS: Numerical Rating Scale; Bold in *P* values represent *P* <0.05.
